# Supplementary material for: DMRforPairs: identifying Differentially Methylated Regions between unique samples using array based methylation profiles
Source: BMC Bioinformatics. 2014 May 15;15:141. doi: 10.1186/1471-2105-15-141 (PMC4046028; doi:10.1186/1471-2105-15-141)
Supplement: Additional file 2 — DMRforPairs output for the comparison of A431-MCF7 and NA17018-NA17105. Please start from the HTML files in each folder. Available via the BMC Bioinformatics website. [file 1471-2105-15-141-S2.zip › 1394847754114233_MOESM2_ESM/A431_MCF7/figures/20422.pdf]

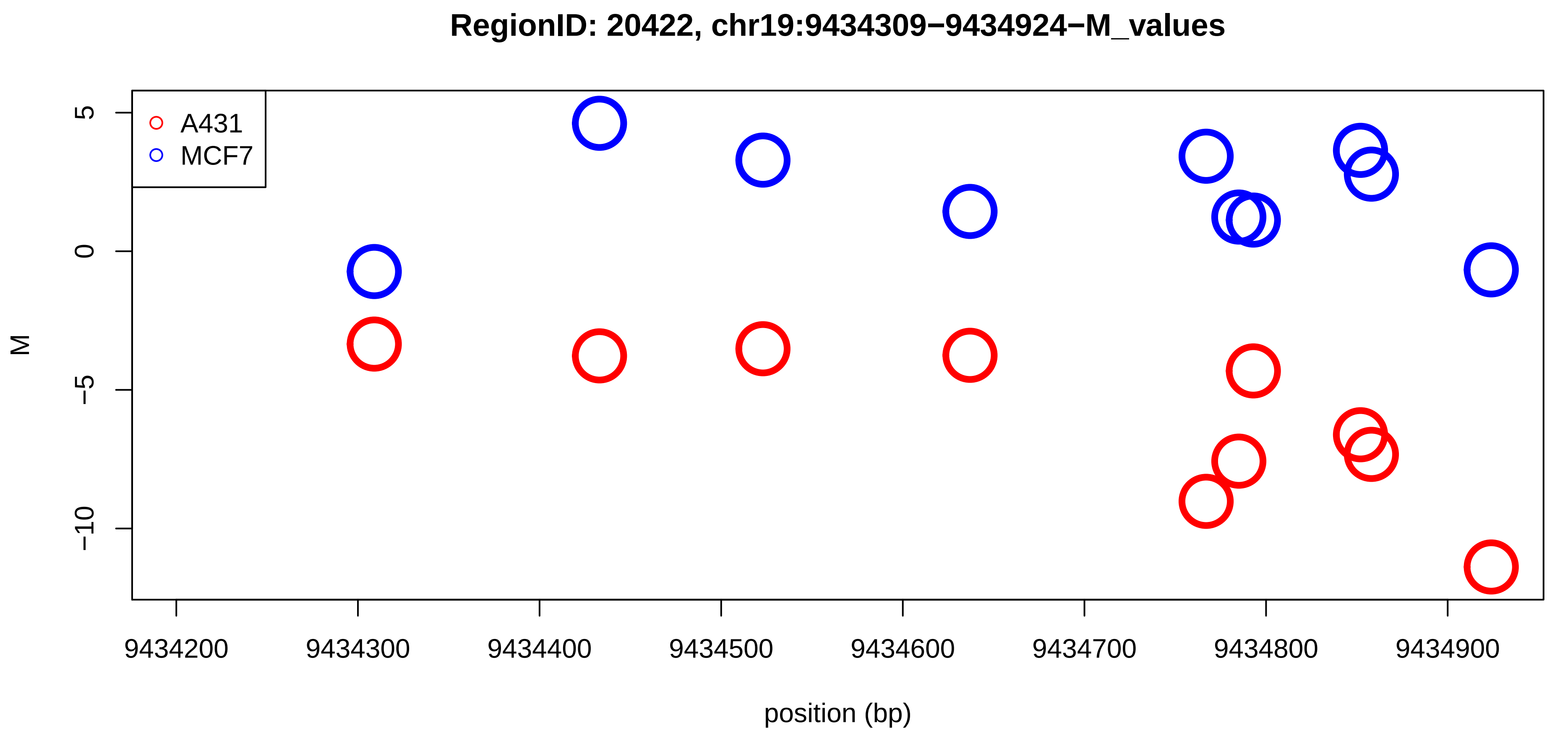
RegionID: 20422, chr19:9434309−9434924−M_values
A431
MCF7
M
5
0
−5
−10
9434200
9434300
9434400
9434500
9434600
9434700
9434800
9434900
position (bp)

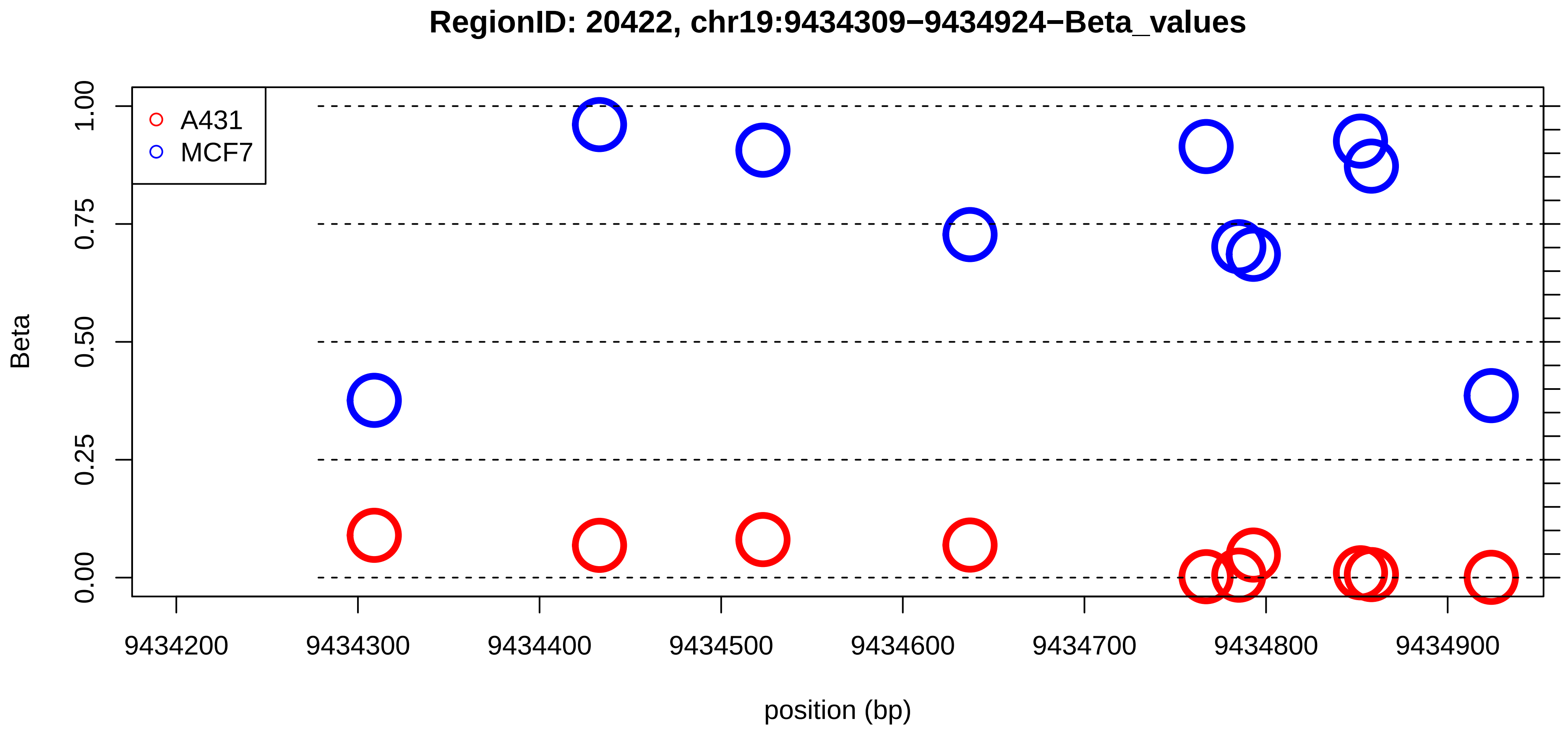
RegionID: 20422, chr19:9434309−9434924−Beta_values
A431
MCF7
Beta
1.00
0.75
0.50
0.25
0.00
9434200
9434300
9434400
9434500
9434600
9434700
9434800
9434900
position (bp)

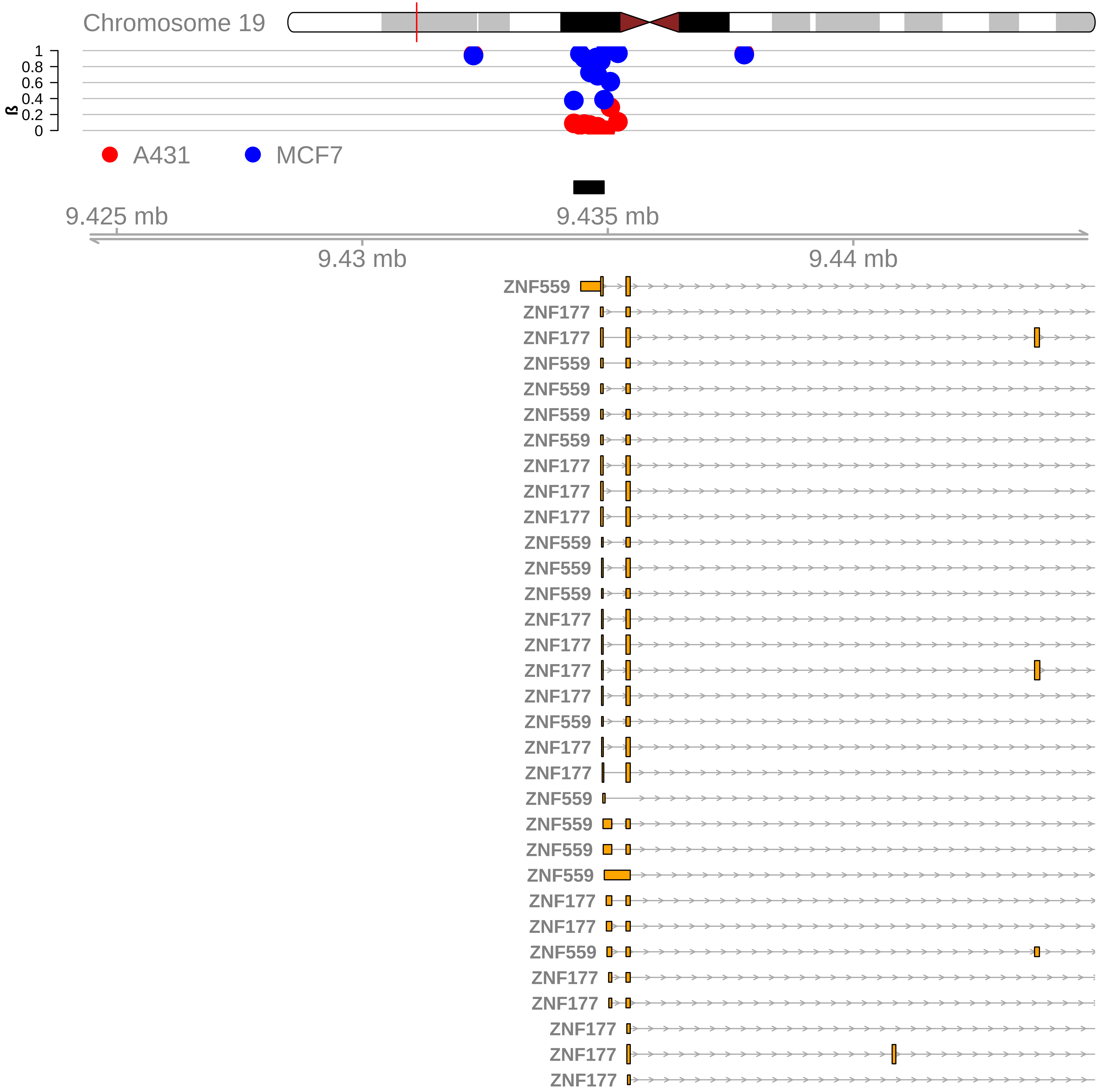

Chromosome 19
β
1
0.8
0.6
0.4
0.2
0
A431
MCF7
9.425 mb
9.435 mb
9.43 mb
9.44 mb
ZNF559
ZNF177
ZNF177
ZNF559
ZNF559
ZNF559
ZNF559
ZNF177
ZNF177
ZNF177
ZNF559
ZNF559
ZNF559
ZNF177
ZNF177
ZNF177
ZNF177
ZNF559
ZNF177
ZNF177
ZNF559
ZNF559
ZNF559
ZNF559
ZNF177
ZNF177
ZNF559
ZNF177
ZNF177
ZNF177
ZNF177
ZNF177
